# Antibody Fragments Directed against Different Portions of the Human Neural Cell Adhesion Molecule L1 Act as Inhibitors or Activators of L1 Function

**DOI:** 10.1371/journal.pone.0052404

**Published:** 2012-12-18

**Authors:** Yan Wang, Gabriele Loers, Hong-Chao Pan, Ricardo Gouveia, Wei-Jiang Zhao, Yan-Qin Shen, Ralf Kleene, Julia Costa, Melitta Schachner

**Affiliations:** 1 Center for Neuroscience, Shantou University Medical College, Shantou, Guangdong Province, People's Republic of China; 2 Zentrum für Molekulare Neurobiologie Hamburg, Universität Hamburg, Hamburg, Germany; 3 Instituto de Tecnologia Química e Biológica, Universidade Nova de Lisboa, Oeiras, Portugal; 4 Keck Center for Collaborative Neuroscience and Department of Cell Biology and Neuroscience, Rutgers University, Piscataway, New Jersey, United States of America; Aix Marseille University, France

## Abstract

The neural cell adhesion molecule L1 plays important roles in neuronal migration and survival, neuritogenesis and synaptogenesis. L1 has also been found in tumors of different origins, with levels of L1 expression correlating positively with the metastatic potential of tumors. To select antibodies targeting the varied functions of L1, we screened the Tomlinson library of recombinant human antibody fragments to identify antibodies binding to recombinant human L1 protein comprising the entire extracellular domain of human L1. We obtained four L1 binding single-chain variable fragment antibodies (scFvs), named I4, I6, I13, and I27 and showed by enzyme-linked immunosorbent assay (ELISA) that scFvs I4 and I6 have high affinity to the immunoglobulin-like (Ig) domains 1–4 of L1, while scFvs I13 and I27 bind strongly to the fibronectin type III homologous (Fn) domains 1–3 of L1. Application of scFvs I4 and I6 to human SK-N-SH neuroblastoma cells reduced proliferation and transmigration of these cells. Treatment of SK-N-SH cells with scFvs I13 and I27 enhanced cell proliferation and migration, neurite outgrowth, and protected against the toxic effects of H_2_O_2_ by increasing the ratio of Bcl-2/Bax. In addition, scFvs I4 and I6 inhibited and scFvs I13 and I27 promoted phosphorylation of src and Erk. Our findings indicate that scFvs reacting with the immunoglobulin-like domains 1–4 inhibit L1 functions, whereas scFvs interacting with the fibronectin type III domains 1–3 trigger L1 functions of cultured neuroblastoma cells.

## Introduction

The cell adhesion molecule L1 (also called L1CAM or CD171), a member of the immunoglobulin superfamily of cell adhesion molecules, plays important roles in cell-cell interactions. In the nervous system [1], [2], L1 is preferentially localized in axons and growth cones of differentiating neurons, supports neural cell migration and survival, and promotes neurite outgrowth, axonal fasciculation [3]–[9], myelination, and synaptic plasticity [10], [11]. Mutations in the X chromosome-localized L1 gene severely affect nervous system functions in affected males, including mental disabilities, aphasia, shuffling gait, and adducted thumbs (MASA syndrome) [12]–[14]. Furthermore, mutations in the L1 gene have also been linked to schizophrenia and Hirschsprung's disease [15]. Besides its functions in the nervous system, L1 plays important roles in tumor progression and metastatis. L1 is expressed in a broad set of tumors comprising not only gastrointestinal stromal tumor, melanoma, neuroblastoma, Schwannoma, paraganglioma, pheochromocytoma of neuroepithelial and neural crest origin [16], but also in tumors of non-neural origin, such as granular cell tumor, chondrosarcoma and Kaposi sarcoma, capillary hemangioma, lymphoblastoma, and cancers of the esophagus, colon, and ovary [17], [18]. Because of its pivotal importance in repair of the nervous system and in the metastatic behavior of tumors, we sought to screen for antibodies that, by reacting with different domains of the human L1 molecule, would, on the one hand, trigger its beneficial functions and, on the other hand, inhibit the detrimental functions of the molecule.

## Materials and Methods

### Expression of L1 fragments in insect cells and subsequent purification by affinity chromatography

Recombinant L1 fragments were produced in *Spodoptera frugiperda* Sf9 cells as described [19]. Briefly, L1 constructs encoding the entire extracellular domain of L1 (L1/ecd) (amino acids 24 to 1108), the immunoglobulin-like domains 1–4 (L1/Ig1–4, amino acids 24 to 425), or the fibronectin type III homologous domains 1–3 (L1/Fn1–3, amino acids 606 to 914) were cloned into the pcDNA3 expression vector and then subcloned into the pMIB-V5-His expression vector (Invitrogen). This expression vector encodes a melittin signal sequence for protein secretion, and V5 and His tags at the C-terminus of the fusion proteins for detection and purification. Pairs of forward/reverse primer sequences for L1/ecd, L1/Ig1–4 and L1/Fn1–3 were 5′TTTGCTAAGCTTGGAGGAATATGAAGGACACCATGTG3′/5′TTAATCCTCGAGCCTCAGTGGCGAAGCCAGC3′, 5′TTTGCTAAGCTTGGAGGAATATGAAGGACACCATGTG3′/5′TTAAACCTCGAGCTGGCAGCTGGACAACGTAGA3′ and 5′CATGCTAAGCTTGCTCTTGGTGGTGGGGAGC3′/ 5′TTTTTGCTCGAGCTGGGGTGCTGAAGGTGAAC3′, respectively. Sf9 insect cells (Life Technologies, Foster City, CA, USA) were transfected with the pMIB-L1/Ig1–4 and pMIB-L1/Fn1–3 plasmids by the calcium phosphate method and pMIB-L1/ecd with Cellfectin (Life Technologies). L1/Ig1–4 and L1/Fn1–3 were produced at 27°C in shaker flasks and L1/ecd was produced in a 2 L bioreactor using Sf900II medium containing 5 µg/ml blasticidin–HCl. Proteins were purified by ion metal affinity chromatography using a HisTrap Sepharose column (GE Healthcare, Piscataway Township, NJ; Fig. S1). Figure S1 shows the purity of the produced L1 fragments (Coomassie stained gel and Western blot with anti-V5 tag antibody (1∶5,000), followed by anti-mouse IgG coupled to horseradish peroxidase (1∶4,000) and detection with Immobilon Western Chemiluminescent HRP Substrate (Millipore) with 1 min exposure). Control without primary antibody did not show any signal.

### Selection of scFvs recognizing extracellular L1 epitopes

The selection of scFvs binding to the extracellular domain (ecd) of human L1 was essentially as described [20]. The Tomlinson I and J libraries (Geneservice, Nottingham, United Kingdom) and the recombinantly expressed L1/ecd were used for screening. Briefly, 100 µl of 22.5 µg/ml L1/ecd in phosphate-buffered saline, pH 7.4 (PBS) were coated overnight at 4°C onto a 96-well tissue culture dish (Jet, biofial, BeiJing, China). Wells were then blocked with 3% BSA (fatty acid free, Merck, Whitehouse Station, NJ, USA) in PBS at room temperature for 1 hour. After washing the wells twice with PBS, 10^13^ phagemid particles in 0.5% BSA in PBS were added to the wells. After incubation for 40 min at room temperature, wells were washed eight times with PBS containing 0.1%, 0.3%, or 0.5% Tween-20 and then rinsed twice with PBS, 5 min each. Bound phages in each well were released by incubation with 100 µl trypsin (Beyotime, Hai Men, China) (10 µg/ml PBS) for 1 hour at room temperature and collected. For amplification, phages were used to infect the *E. coli* strain TG1. Bacteria were grown at 37°C overnight on TYE plates (10 g Bacto-tryptone, 5 g Bacto-yeast extract, and 8 g NaCl in 1 L distilled water, pH 7.4) containing 100 µg/ml ampicillin and 1% glucose. After three rounds of panning, individual phage clones were selected for ELISA. For phage ELISA, each well of a 96-well plate was coated overnight at 4°C with 100 µl of 10 µg/ml L1/ecd in PBS, and blocked with 3% BSA in PBS for 1 hour at room temperature. Supernatants from individual clones were added to the wells, incubated at room temperature for 40 min and washed three times with PBST (PBS, 0.1% Tween 20). Wells were then incubated with a 1∶3,000 dilution of the monoclonal mouse anti-M13 horseradish peroxidase (HRP) conjugated antibody (GE Healthcare) in 3% BSA in PBS for 1 hour at room temperature and washed three times with PBST. Binding of phages was detected using TMB (3, 3′, 5, 5′-tetramethylbenzidine; Beyotime) as a substrate for the HRP.

### Sequencing of phagemid DNA

The sequences of selected clones were determined with the primer LMB (5′-CAG GAA ACA GCT ATG AC-3′) by the dideoxy chain terminating method. Sequencing was repeated three times for verification. Sequence data were analyzed using the DNA Star program (DNASTAR software).

### Purification of scFvs

**Figure 1:**
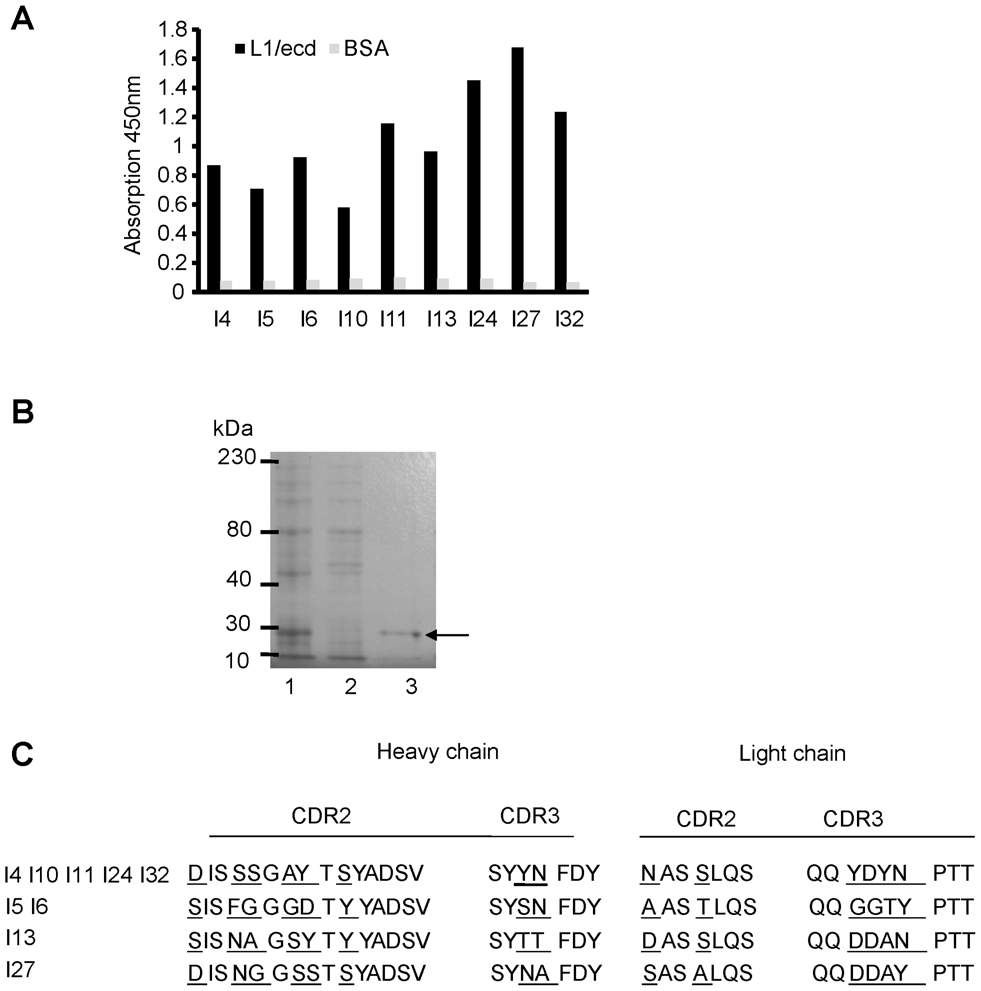
Selection and purification of L1 scFvs from the Tomlinson I library. (**A)** Binding of monoclonal phages to substrate-coated L1/ecd was determined by ELISA. Phages (1×10^9^ cfu) from the third selection round were incubated with L1/ecd. Substrate-coated BSA was used as negative control. Bound phages were detected with anti-M13 antibody conjugated to HRP. (**B**) Expression and purification of scFvs. Lane 1, bacterial supernatant containing scFvs; lane 2, column flow-through; lane 3, affinity chromatography purified scFv I27 (indicated by arrow) following column elution with 100 nM imidazole. Molecular weight markers are indicated at the left margin. (**C**) Amino acids sequences of complementarity determination regions 2 and 3 on heavy and light chains of L1 scFvs. Different amino acids are underlined.

The phagemid clones were amplified and phages were extracted as described [20]. For the production of soluble scFv proteins, 1.0 ml inocula of *E. coli* HB 2151 non-suppressor strain infected with a glycerol stock of an individual phage-ScFv clone was transferred into culture flasks containing 1 L 2×TY/100 µg/ml ampicillin/0.1% glucose. The culture was grown with constant shaking (250 rpm) at 37°C until the OD_600 nm_ was approximately 0.9. At this stage, expression of the scFv cassette was induced by isopropyl β-D-thiogalactopyranoside (IPTG), which was added to the culture in 2×TY containing 100 µg/ml ampicillin to give a final concentration of 1 mM IPTG. Shaking was continued at 30°C and 200 rpm overnight. ScFvs secreted into culture supernatant and the *E. coli* periplasm were harvested after osmotic shock [20]. Supernatants were then centrifuged at 10,000×g at 4°C for 30 min and clarified by filtration through 0.22 µm filters (PALL, Port Washington, NY, USA). Finally, all clarified protein fractions (supernatant and periplasmic fraction) were pooled and passed through a His trap column (GE Healthcare) and dialyzed against PBS. Purity of the eluted soluble scFvs was evaluated by SDS–PAGE on 10% gels (Fig. 1). The concentration of the purified scFvs was determined by the Beyotime BCA technique.

### Characterization of purified scFvs by ELISA

**Figure 2:**
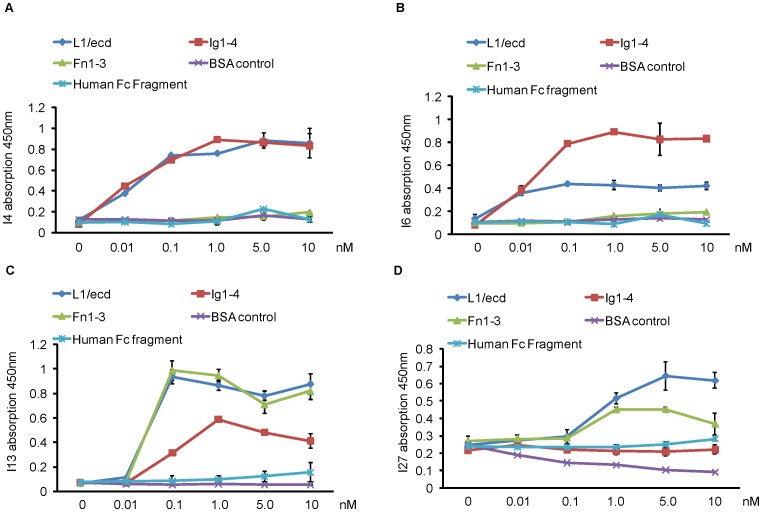
Binding of purified scFvs to L1. Binding of purified scFvs to L1/ecd, Ig1–4, Fn1–3 was determined by ELISA. (**A**) ScFv I4, (**B**) scFv I6, (**C**) scFv I13, (**D**) scFv I27. ScFvs were incubated with substrate-coated L1/ecd, Ig1–4, Fn1–3, human Fc fragment, or bovine serum albumin (BSA) at the indicated concentrations. Bound scFvs were detected with biotinylated antibodies against c-myc followed by incubation with ExtrAvidin-HRP. Data represent mean values ± SEM from 3 independent experiments.

Ninety-six-well plates were coated overnight at 4°C with 100 µl L1/ecd, L1/Fn1–3, L1/Ig1–4 in PBS (Fig. 2A) at concentrations of 0–10 nM. Wells were blocked with 3% BSA in PBS for 1 hour at room temperature. Individual scFvs (100 µl, 100 ng/ml in PBS containing 3% BSA) were added to the wells, incubated at room temperature for 40 min, and washed with 0.1% PBST three times, 5 min each. Wells were then incubated with biotin-conjugated mouse anti-c-myc monoclonal antibody 9E10 (Sigma-Aldrich, St. Louis, MO, USA) for 1.5 hours at room temperature, washed three times with 0.1% PBST, and then incubated with ExtrAvidin-HRP (Sigma-Aldrich) for 1 hour. Wells were washed, and binding was detected using TMB as a substrate.

### Human SK-N-SH neuroblastoma cell culture

Human SK-N-SH cells (CCTCC, China Center for Type Culture Collection, Shang Hai, China), which were shown to express L1 [21], were cultured at 37°C and 5% CO_2_ in DMEM supplemented with 10% fetal bovine serum, 2 mM L-glutamine, 100 U/ml penicillin and 100 µg/ml streptomycin (Invitrogen, Carlsbad, CA, USA).

### Immunofluorescence assay

For staining of live SK-N-SH cells, cells were pre-blocked with 5% fetal bovine serum in PBS for 15 min at room temperature. ScFvs were then added to a final concentration of 20 µg/ml, and cells were incubated for 30 min at room temperature. After washing, cells were fixed for 5 min in 4% paraformaldehyde (PFA) and washed again with PBS, followed by rabbit anti-His tag (diluted 1∶200, Biosis, Bei Jing, China) or goat anti-human L1/ecd (diluted 1∶300, R&D Systems, Minneapolis, MN, USA) and incubation for another 30 min at room temperature. Antibody binding was detected by incubation for 1 hour at room temperature with Alexa 488-conjugated donkey anti-rabbit IgG (Jackson Laboratories, Bar Harbor, ME, USA) or Alexa 594-conjugated donkey anti-goat IgG (Jackson Laboratories). For control, cells were treated in the same manner, except that scFvs were omitted. For Ki67 staining, SK-N-SH cells were fixed with 4% PFA, washed, permeabilized for 5 min with 0.1% Triton-X 100 in PBS, and incubated overnight at 4°C with rabbit anti-human Ki67 (Roche, South San Francisco, CA, USA), followed by Alexa 594-conjugated donkey anti-rabbit IgG (diluted 1∶400, Jackson Laboratories).

### Proliferation and cell viability/survival assays

Cells were seeded in serum free DMEM medium (Sigma/Aldrich) into 96-well plates at a density of 5×10^3^ cells per well and treated with scFvs (0.033–16.5 µM) for 24 or 48 hours in an incubator (5% CO_2_ and 37°C). Proliferation and cell survival were assessed using a Cell Count Kit-8 (MST-8) (Beyotime). A non-immune human IgG (Jackson Laboratories) treated group was used as control. To evaluate the response of cells to oxidative stress, cells were seeded in serum free DMEM medium (Sigma/Aldrich) into 96-well plates at a density of 10^4^ cells per well. Twelve hours after seeding, cells were treated with H_2_O_2_ (250 µM) for 2 hours in an incubator (5% CO_2_ and 37°C). After 2 hours the medium was changed to fresh serum free culture medium, and cells were treated with of scFvs (10–300 nM) and further maintained for 12 or 24 hours (5% CO_2_ and 37°C).

### Cell transmigration assay

Twenty-four well transwell plates, 8 µm pore size (Transwell, Costa, Corning, Bei Jing, China), were used to assay cell migration. SK-N-SH cells (2×10^5^ or as otherwise indicated) in DMEM containing 0.5% BSA were inoculated into the upper chamber of the plates and allowed to migrate to the lower chamber containing 16.5 µM scFvs over a 36-hour period at 37°C and in 5% CO_2_. To quantify transmigrated cells, the upper chamber was removed and cleaned carefully with a cotton swab to remove the non-migrated cells. Migrated cells adherent to the bottom of the membrane of the upper chamber were stained with 0.1% crystal violet solution. The membrane was extensively washed in water and dried under air. The number of migrated cells was calculated by scoring six random fields per membrane as viewed with an Olympus BX51 microscope at 400× magnification. Cells in the upper chamber were counted to check for effects of scFvs on proliferation that could indirectly influence the number of transmigrated cells.

### Neurite outgrowth assay

Human SK-N-SH cells were seeded at a density of 2×10^3^ per well in 100 µl culture medium into 96-well tissue culture plates coated with poly-D-lysine (PDL) and 0.1–3 µM scFvs, 3 µM L1/ecd or 10 µM IgG. After 5 days cells were fixed by adding 10 µl 25% glutaraldehyde to each well, and cells were stained with Toluidine blue (2.5% in 1% sodium tetraborate). Neurite lengths of individual cell were determined by the 400× Image Analysis System (Olympus). Only neurites that did not contact other cells and had a length of at least one cell body diameter were measured.

### Hoechst 33342 and PI staining

Twelve hours after seeding on coverslips in 24 well-plates, cells were treated with H_2_O_2_ (250 µM) for 2 hours in an incubator (5% CO_2_ and 37°C). After 2 hours the medium was changed to fresh serum free culture medium and cells were treated with different concentrations of scFvs and further maintained for 24 hours (5% CO_2_ and 37°C). Cells were washed with PBS, followed by staining with the double staining kit containing Hoechst 33342, propidium iodide (PI) and cell staining buffer (Beyotime).

### Western blot analysis

1×10^6^ cells were collected by centrifugation and incubated for 20 min in twice the pellet volume with ice-cold buffer containing 1.5 mM MgCl_2_, 10 mM KCl, 10 mM Tris–HCl pH 7.9, 3 mM dithiothreitol (DTT), 0.1% NP-40 (Beyotime) and a protease inhibitor cocktail (Beyotime). Twenty five µg protein was subjected to 12% SDS-PAGE, transferred onto a PVDF membrane (Millipore, Temecula, CA, USA), and probed with rabbit anti-human src (Santa Cruz Biotechnology, Santa Cruz, CA, USA), mouse anti-phospho-src (Santa Cruz Biotechnology), mouse anti-phospho-Erk1/2 (Beyotime), rabbit anti-human Erk1/2 (Beyotime), rabbit anti-human Bcl-2 (Biosis, Bei Jing, China), mouse anti-human Bax, mouse anti-human GAPDH (glyceraldehyde-3-phosphate dehydrogenase) (BOSTER, Wu Han, China). The enhanced chemiluminescence system (Beyotime) was used for detection of immunoreactive proteins with horseradish peroxidase (HRP)-conjugated IgG as secondary antibodies (BOSTER). Intensity of immunostaining was measured with Image J software.

### Statistical analysis

The significance of values was determined by Student's t-test (two-tailed) test, except for the experiments measuring oxidative stress under H_2_O_2_, for which one-way ANOVA with Dunnet's post-hoc test was used. Values are expressed as means ± SEM from at least three independent experiments and differences were considered significant at *p*<0.05.

## Results

### Selection and purification of L1 scFvs against the L1/ecd

Two phagemid libraries (Tomlinson I and J) were screened for phages/scFvs binding to the human L1/ecd. Only from the Tomlinson I library scFvs reacting with the human L1/ecd could be obtained. Positive clones were selected by phage ELISA using substrate-coated L1/ecd as target and BSA as negative control (Fig. 1A). ScFvs were purified by nickel column chromatography and subjected to SDS-PAGE (Fig. 1B). DNA extracted from individual positive clones was sequenced and analyzed for amino acid sequences in the complementarity determining region 2 and 3 of the heavy chain and light chains (Fig. 1C). Clone I4 was found 5 times, I6 two times, and I13 and I27 only once. For subsequent investigations, we selected four positive clones I4, I6, I13, I27 which showed the strongest binding to L1/ecd and which did not bind to BSA.

### ScFvs I4 and I6 bind to the Ig domains 1–4, and L1 scFvs I13 and I27 bind to the Fn domains 1–3

To narrow down to which domains of L1/ecd the scFvs bind, L1/ecd, L1/Ig1–4, and L1/Fn1–3 were used as substrates. None of the four scFvs reacted with the substrate-coated human IgG Fc fragment, which served as negative control (Fig. 2A–D). The scFvs bound to the positive control substrate L1/ecd in a concentration dependent manner. Both scFvs I4 (Fig. 2A) and I6 (Fig. 2B) showed strong binding to L1/Ig1–4 in the higher concentration range of 1.0 nM to 10 nM and did not bind to L1/Fn1–3. ScFv I13 reacted with L1/Fn1–3 in a concentration range of 0.1 nM to 10 nM and weakly bound to L1/Ig1–4, whereas binding of I13 to L1/Fn1–3 was stronger than its binding to L1/Ig1–4 (Fig. 2C). ScFv I27 (Fig. 2D) reacted strongly with L1/Fn 1–3 in a concentration range of 0.5 nM to 10 nM and did not bind to L1/Ig1–4. We thus consider scFvs I4 and I6 to bind to Ig1–4 in a specific manner, and scFv I27 to specifically react with Fn1–3. The reason why scFv I13 reacted not only with Fn1–3, but also with Ig1–4 at higher concentrations, is presently not understood, but may be caused by identical amino acid stretches in the Fn1–3 and Ig1–4 domains of L1.

The L1 fragments used for the binding are probably N-glycosylated since the estimated molecular mass by SDS-PAGE is higher (Fig. S1) than that predicted from the amino acid sequence (130, 49 and 39 kDa for L1/ecd, L1/Ig1–4 and L1/Fn1–3, respectively). L1 fragments from Sf9 cells carry glycosylation of the paucimannosidic type as described [22], being distinct from human L1 glycosylation (NT2N neurons) that comprises complex glycans (unpublished results) and also the sulfated HNK-1 glycan [23]. Paucimannosidic structures are not usually found at the surface of human cells. Therefore, the binding of the selected scFvs and their biological effects (below) should not involve the L1 glycans.

### ScFvs bind to L1 at the cell surface of human neuroblastoma SK-N-SH cells

**Figure 3:**
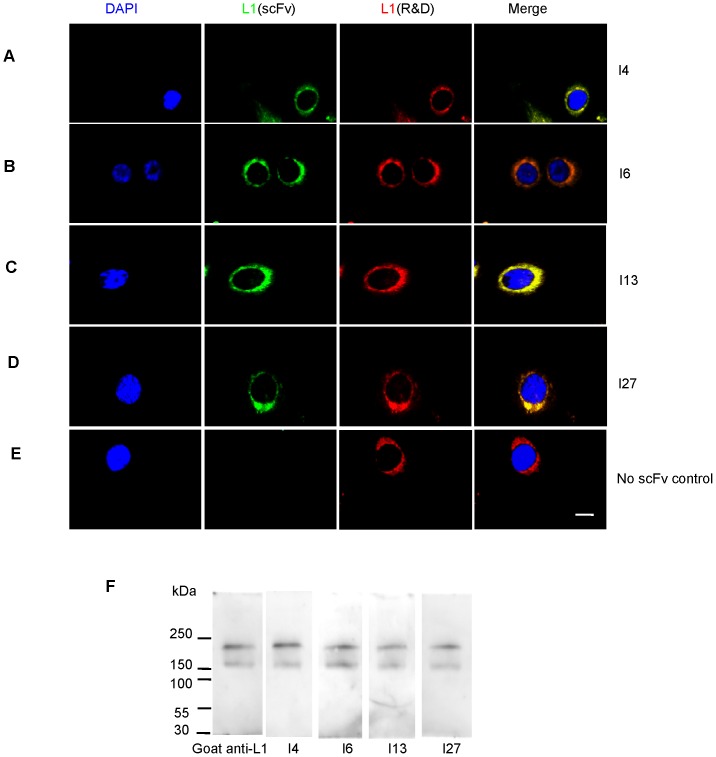
Immunostaining of live SK-N-SH cells with ScFvs. Substrate-attached SK-N-SH cells were incubated with scFvs I4 (**A**), I6 (**B**), I13 (**C**), or I27 (**D**), or without scFv (**E**). Bound scFvs were visualized by rabbit antibody against the His-tag followed by incubation with Alexa 488 nm (green)-conjugated goat anti-rabbit IgG and double labeling with goat anti-human extracellular domain of L1 as positive control, followed by Alexa 594 nm (red)-conjugated donkey anti-goat IgG. Bar in (**E**) indicates 5 µm for all panels. (**F**) Binding of purified scFvs I4, I6, I13, I27 to an SK-N-SH cell lysate was tested by subjecting 50 µg protein to SDS-PAGE in 8% gels under reducing conditions. Western blot analysis was carried out with scFvs I4, I6, I13, I27, and goat anti-human L1 as primary antibodies. Primary antibodies were detected with secondary antibody against His or rabbit anti-goat, respectively. Molecular weight markers are indicated at the left margins in kilodaltons (kDa).

To determine the ability of scFvs to bind to native L1 protein at the cell surface, immunostainings of live neuroblastoma cells were performed. Stainings with scFvs and goat anti-human L1/ecd antibody showed co-localization of L1 immunoreactivities using commercial anti-L1 antibody and individual scFvs directed against the L1/ecd (Figs. 3A–D), indicating that the scFvs recognize L1 at the cell surface. No immunostaining was observed when the scFv antibodies were omitted (Fig. 3E). We previously identified scFvs against mouse Fn1–2 [24] that do not cross-react with mouse NCAM or CHL1 (close homolog of L1) which are homologous to L1. The members of the L1 family share most homologies in their intracellular domains (for instance L1 and CHL1) and we have found that polyclonal and monoclonal antibodies to the extracellular domains do not cross-react (unpublished data). ScFvs were also probed with SK-N-SH cell lysates by Western blot analysis, showing that the scFvs reacted with L1 forms of 220 and 200 kDa (Fig. 3F) which represent the full-length L1 and an L1 fragment resulting from proteolytic cleavage of full-length L1. Thus, scFvs I4, I6, I13 and I27 react with native L1 protein *in vitro*.

### ScFvs I4 and I6 inhibit, while scFvs I13 and I27 enhance proliferation of SK-N-SH cells

**Figure 4:**
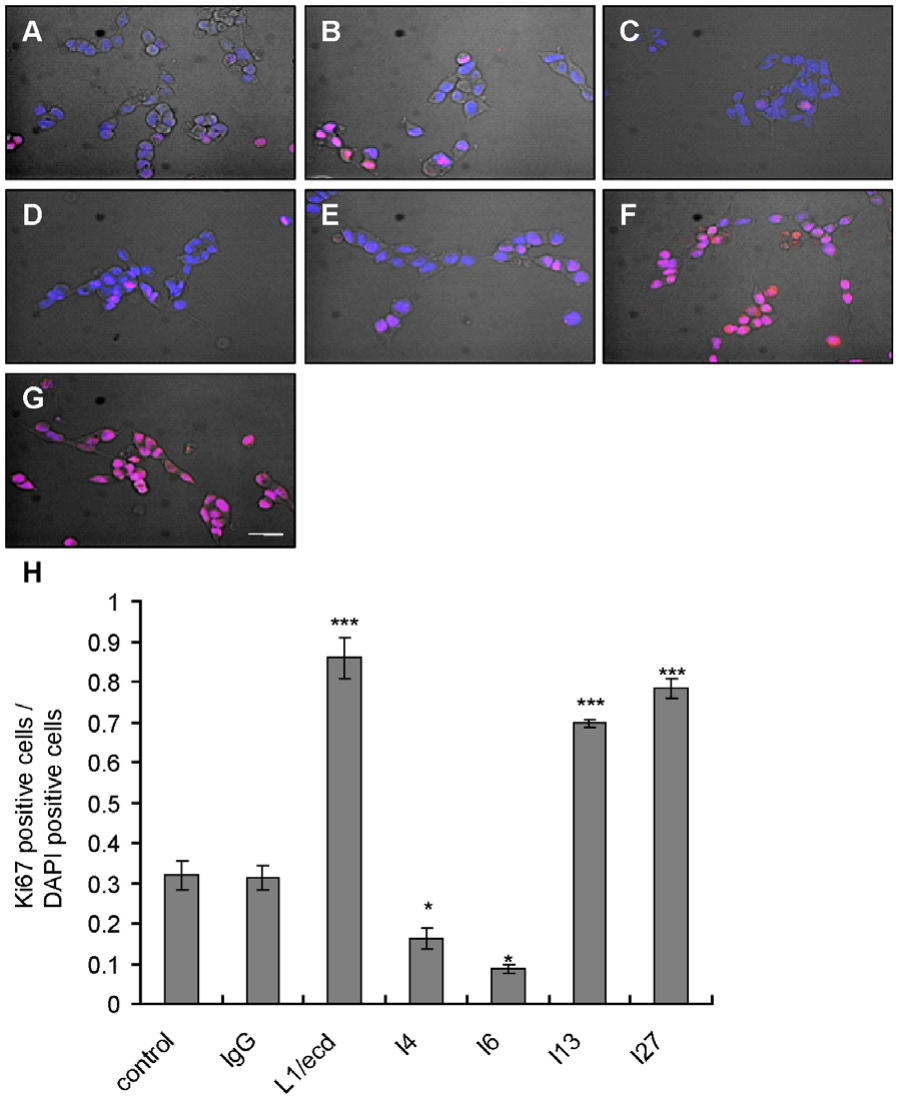
ScFvs against Ig1–4 inhibit, while scFvs against Fn1–3 increase proliferation of SK-N-SH cells. Representative images are merged with bright field, DAPI (blue) nuclear staining, and Ki67 immunostaining (red). (**A**) Control without treatment, (**B**) negative control with non-immune human IgG (10 µM), (**C**) scFv I4, (**D**) scFv I6, (**E**) scFv I13, (**F**) scFv I27, (**G**) positive control with L1/ecd (all at 16.5 µM). Scale bar in (**E**) indicates 20 µm (for all panels). (**H**) Mean values ± SEM of the ratio of Ki67-immunopositive cells to DAPI-positive cells are shown from three independent experiments. Asterisks denote significant differences from control. *** *p<*0.001, ** *p<*0.01, * *p*<0.05, Student's t-test.

To investigate the effect of scFvs on cell proliferation, the number of SK-N-SH cells was determined after treatment with different concentrations of scFvs, with non-immune human IgG as negative control, and with L1/ecd as positive control for 24 to 48 hours. ScFvs I4 and I6 reduced the number of proliferating SK-N-SH cells in a dose- and time-dependent manner, whereas scFvs I13 and I27 enhanced the number of proliferating cells. Non-immune human IgG did not show any effect on SK-N-SH cell numbers (data not shown). The number of SK-N-SH cells which were immunopositive for the proliferation marker Ki67 was dramatically decreased after treatment with I4 and I6 for 48 hours (Fig. 4C, D), when compared to the cell number obtained after treatment with non-immune IgG or untreated cells (Fig. 4A, B), whereas treatment with I13 and I27 increased the number of Ki67-immunopositive cells compared to controls (Fig. 4E, F). L1/ecd also increased the level of Ki67 immunoreactivity (Fig. 4G). ScFvs I4 and I6 binding to L1/Ig1–4 inhibit, while I13 and I27 binding to L1/Fn1–3 promote proliferation of SK-N-SH cells (Fig. 4H).

### ScFvs I4 and I6 inhibit, whereas scFvs I13 and I27 promote migration of SK-N-SH cells

**Figure 5:**
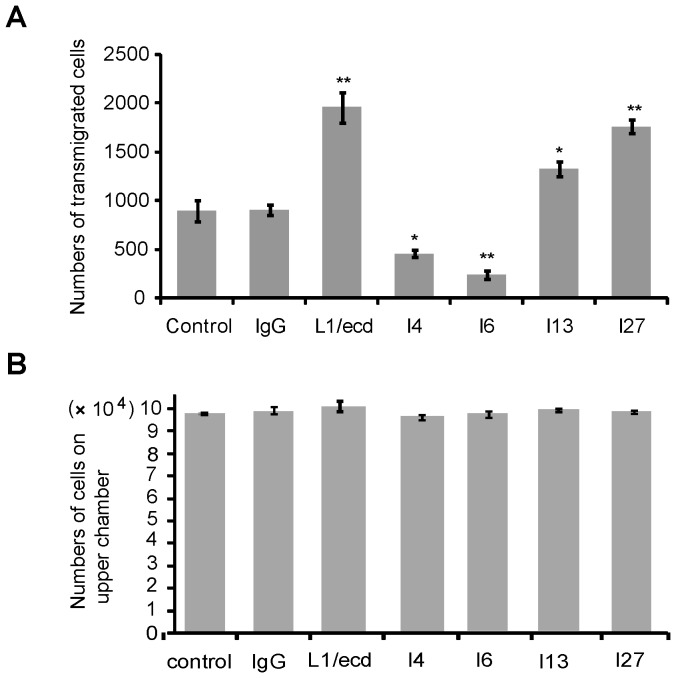
ScFvs against Ig1–4 inhibit, while scFvs against Fn1–3 stimulate transmigration of SK-N-SH cells. Equal numbers of cells (10^5^) were seeded into the upper compartment of a transwell chamber and allowed to migrate through a BSA-coated membrane for 36 hours. (**A**) Cells that had migrated into the lower chamber containing L1/ecd (12 µM), non-immune human IgG (10 µM), and scFvs I4 (16.5 µM), I6 (16.5 µM), I13 (16.5 µM), or I27 (16.5 µM) are shown. Omission of scFvs served as negative control. Migrated cells were fixed on the membrane and lower chamber, stained with crystal violet and counted. (**B**) Non-transmigrated cells in the upper chamber were also counted. Data represent mean values ± SEM of numbers of transmigrated cells from three independent experiments. Asterisks denote significant differences from control. ** *p*<0.01, * *p*<0.05 versus the number of cells in the control, Student's t-test.

To determine whether scFvs also affect cell migration, we performed a transmigration assay with SK-N-SH cells. In the presence of scFvs I4 and I6, the number of migrated cells was reduced in comparison to the number determined in the presence of non-immune IgG control (Fig. 5A). In the presence of scFvs I13 and I27, the number of transmigrating cells was increased relative to the number in the presence of non-immune IgG control (Fig. 5A). To exclude the possibility that proliferation would affect the numbers of transmigrated cells, non-transmigrated cells were counted in the upper chamber showing that there were no differences in cell numbers of cells in the presence or absence of scFvs (Fig. 5B). These results show that transmigration of SK-N-SH cells is inhibited by scFvs I4 and I6, but enhanced by scFvs I13 and I27.

### ScFvs I4 and I6 inhibit, while I13 and I27 promote neurite outgrowth of SK-N-SH cells

**Figure 6:**
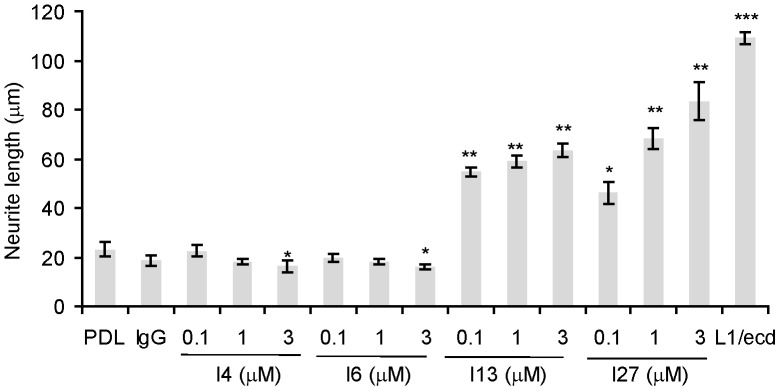
ScFvs against Ig1–4 inhibit, while scFvs against Fn1–3 stimulate neurite outgrowth from SK-N-SH cells. Human SK-N-SH cells were seeded into poly-D-lysine (PDL)-pretreated culture plates coated with human non-immune human IgG (10 µM), L1/ecd (3 µM), scFvs I4, I6, I13 and I27 as substrates at the indicated concentrations and cultured at 37°C. Cultures were fixed after 5 days and stained with toluidine blue. The lengths of neurites were measured. Data represent mean values of neurite lengths per cell ± SEM as compared with wells coated with PDL only from three independent experiments. Asterisks denote significant differences from control. *** *p*<0.001, ** *p*<0.01, * *p*<0.05, Student's t-test.

Previous data have shown that monoclonal L1 antibody 557 directed against an epitope within the Fn3 domain of murine L1 enhances neurite outgrowth [25] and that scFvs directed against the Fn1–2 domain of murine L1 increase neurite lengths of cultured primary cerebellar neurons [24]. We therefore asked whether scFvs directed against Fn1–3 of human L1 can also stimulate neurite outgrowth. We coated scFvs as substrate and measured the length of neurites per SK-N-SH cell. On substrate-coated scFvs I13 and I27, the lengths of neurites were significantly longer compared to neurites on substrate-coated PDL (Fig. 6). Since it was shown that the Ig1–2 domains of L1 trigger neurite growth through binding to integrin [26], we additionally treated cells with scFvs I4, I6 and measured the length of neurites. Both, scFvs I4 and I6, which bind to the Ig1–4 domain of L1, reduced neurite outgrowth when applied as substrate-coat at 3 µM concentration (Fig. 6). As expected, enhanced neurite outgrowth was obtained by treatment of cells with L1/ecd as a positive control (Fig. 6). These results indicate that scFvs against the Ig1–4 domain of L1 inhibit, while scFvs against the Fn1–3 domain of L1 promote neurite growth.

### ScFvs I13 and I27 reverse the toxic effects of hydrogen peroxide on SK-N-SH cells

**Figure 7:**
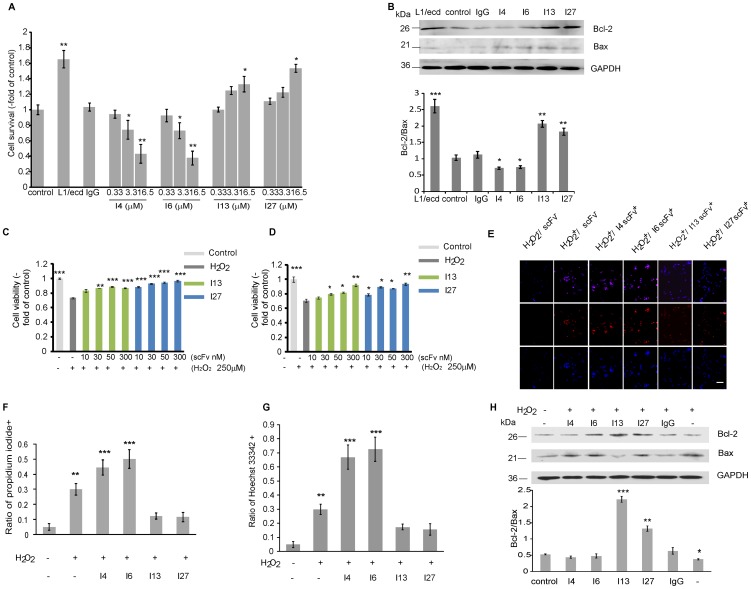
ScFvs against Fn1–3 protect cells from H_2_O_2_–induced death and increase the ratio of Bcl-2/Bax. SK-N-SH cells were seeded into poly-D-lysine (PDL)-pretreated culture plates coated with L1/ecd (as positive control), non-immune human IgG (as negative control), and scFvs I4, I6, I13, or I27 and maintained for 5 days in serum-free medium. In addition, SK-N-SH cells grown for 12 hours were pre-incubated with 250 µM H_2_O_2_ for 2 hours in serum-free medium. After removal of H_2_O_2_, scFvs I13 and I27 were added at the indicated concentrations and cells were cultured for additional 24 hours. (**A**) ScFvs treatment for 5 days. Cell survival was measured by the MST-8 assay. (**B**) L1/ecd, non-immune human IgG, scFvs I4, I6, I13 or I27 (all at 16.5 µM) treatment for 24 hours for Western blot assay of Bcl-2 and Bax protein expression. (**C**) ScFv treatment for 12 hours. (**D**) ScFv treatment for 24 hours. (**C, D**) Cell viability was measured by the MST-8 assay. Data represent mean values ± SEM from four independent experiments. Asterisks denote significant differences from control. *** *p*<0.001, ** *p*<0.01, * *p*<0.05 versus H_2_O_2_ treatment alone, one-way ANOVA, Dunnet's post-hoc test. (**E**) ScFvs I4, I6, I13 and I27 (all at 16.5 µM), were added after removal of H_2_O_2_ for 24 hours. Cells were stained by Hoechst 33342 (blue) and propidium iodide (PI, red), merge (pink). Bar indicates 40 µm for all panels. (**F**, **G**) Quantification of PI and Hoechst positive cells, and calculation of the number of PI and Hoechst positive cells among the total cell number. (**B**, **H**) Analysis of protein levels of Bcl-2/Bax after treatment of cells with scFvs. GAPDH was used as loading control. (**B**, **E**, **F**, **H**) Data represent mean values ± SEM from three independent experiments. *** *p*<0.001, ** *p*<0.01, * *p*<0.05, Student's t-test.

Since it had been shown that scFvs reacting with the Fn1–2 domain of mouse L1 prevent cell death of cultured mouse cerebellar neurons [24], we performed a cell death analysis and determined the survival of cells treated by scFv I4, I6, I13 or I27 for 7 days. ScFvs I4 and I6, which bind to Ig1–4 of L1, reduced cell survival at a concentration of 16.5 µM (Fig. 7A) and reduced the ratio of Bcl-2/Bax (Fig. 7B), while scFvs I13 and I27, which bind to Fn1–3 of L1, promoted cell survival (Fig. 7A) and enhanced the ratio of Bcl-2/Bax (Fig. 7B). We assessed if scFvs I13 and I27 that react with Fn1–3 are also able to protect cells against oxidative stress. Therefore, SK-N-SH cells were exposed to 250 µM hydrogen peroxide for 2 hours followed by application of scFvs I13 and I27 for 12 and 24 hours. The number of dead cells was reduced after addition of the scFvs relative to the number determined in the presence of non-immune human IgG (Fig. 7C, D), indicating that both scFvs protect SK-N-SH cells from cell death. We also found less Hoechst 33342/PI positive cells after treatment with scFvs I13 and I27 than in the absence of scFvs or in the presence of scFvs I4 and I6 after 24 hours (Figs. 7E–G). ScFvs I13 and I27 increased the ratio of Bcl-2/Bax also after hydrogen peroxide treatment (Fig. 7H). These results show that scFvs I4 and I6 treatment reduced cell survival, while treatment of cells with scFvs I13 and I27 promoted cell survival and prevented hydrogen peroxide induced cell death of SK-N-SH cells through Bcl-2.

### ScFvs affect phosphorylation of src in SK-N-SH cells

**Figure 8:**
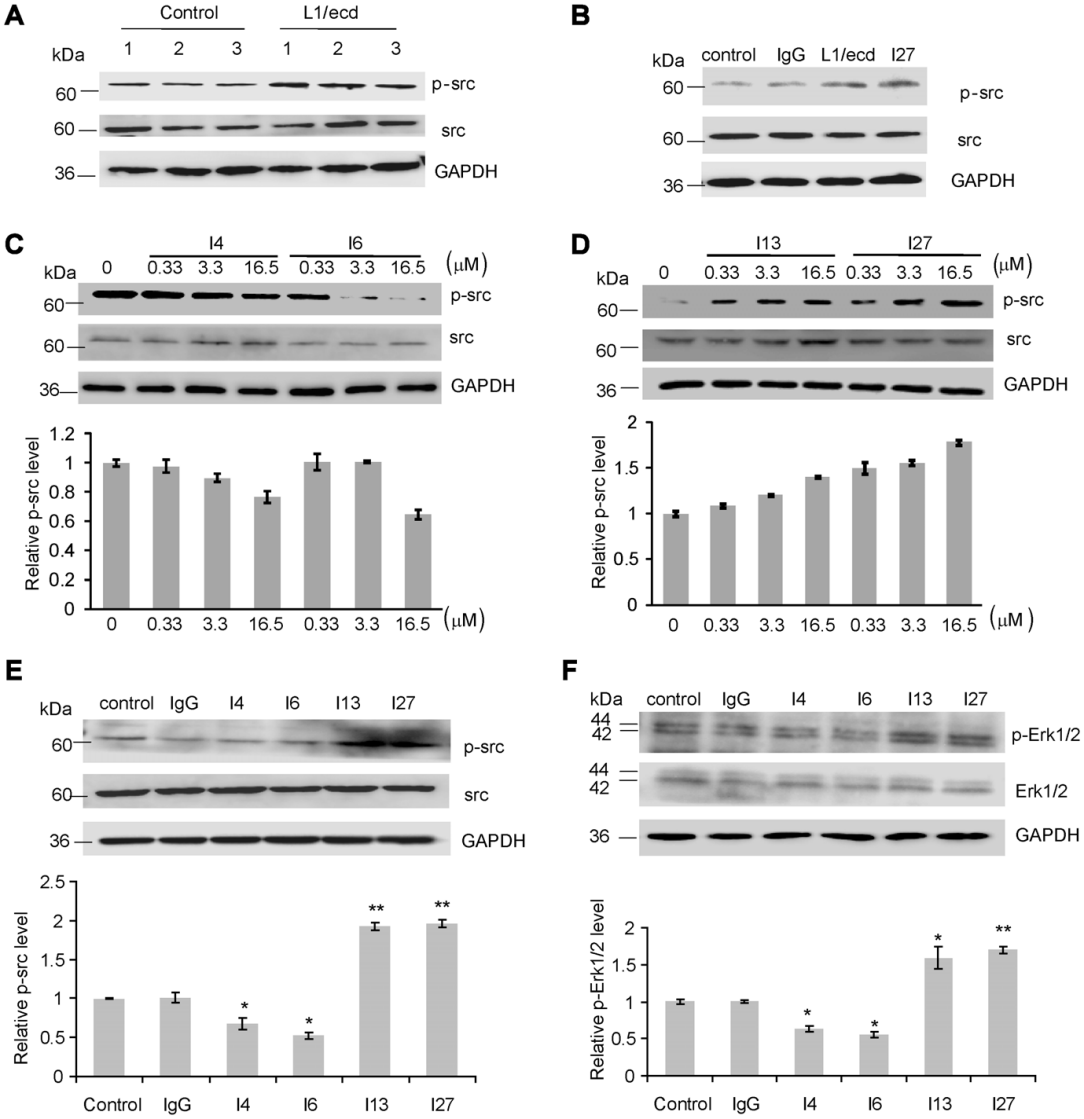
ScFvs against Ig1–4 reduce, while scFvs against Fn1–3 enhance levels of phospho-src and downstream phospho-Erk1/2. Cultured SK-N-SH cells were incubated with L1/ecd (as positive control), non-immune human IgG (as negative control), and scFvs I4, I6, I13, or I27 for 30 min in serum-free medium. (**A**) Levels of src and phospho-src of cells treated with L1/ecd (at 16.5 µM, right) or untreated (control, left), with lanes 1, 2 and 3 showing representative blots from three independent experiments with L1/ecd treated or untreated cells. (**B**) Src and phospho-src levels of cells treated with non-immune human IgG, L1/ecd or I27 (all at 16.5 µM) or left untreated (control). (**C**) Src and phospho-srclevels of cells treated with the indicated concentrations of scFv I4 or I6. (**D**) Src and phospho-src levels of cells treated with the indicated concentrations of scFv I13 or I27. (**E–F**) Comparison of levels of src and phospho-src (**E**) and Erk1/2 and phospho-Erk1/2 (**F**) in untreated cells (control) or cells treated with IgG, scFv I4, I6, I13 or I27 (all at 16.5 µM). Representative images from three independent experiments are shown in **A–E**. For the quantification shown in **C–F**, values of phospho-src (**C–E**) and phospho-Erk1/2 (**F**) were normalized to GAPDH as loading control. Mean values ± SEM from three independent experiments are shown. Asterisks denote significant differences from control. ** *p*<0.01, * *p*<0.05 by Student's t-test.

L1 interacts with integrins or itself and stimulates a downstream pathway through src and Erk [26], [27]. As expected for positive control, L1/ecd stimulated phosphorylation of src under the conditions of the present study (Fig. 8A, B). ScFvs I4 and I6 against Ig1–4 reduced levels of phospho-src in a dose-dependent manner (Fig. 8C), whereas phospho-src levels were elevated by treatment with scFvs I13 and I27 in a dose-dependent manner (Fig. 8D). We also determined levels of both phospho-src (Fig. 8E) and phospho-Erk1/2 (Fig. 8F) after scFv treatment and found that levels of phospho-src and phospho-Erk1/2 were reduced by scFvs I4 and I6 (at a concentration of 16.5 µM) compared with the non-treated control, whereas the levels were increased by scFvs I13 and I27. Phospho-src and phospho-Erk1/2 levels were not influenced by non-immune IgG which served as a negative control.

## Discussion

### Two functionally distinct types of scFvs react with human L1

We identified two scFvs (I4 and I6) directed against Ig1–4 within the extracellular domain of human L1 and two scFvs (I13 and I27) directed against the Fn1–3 domain. The scFvs recognized L1 at the cell surface of cultured neuroblastoma cells, but showed opposite functional effects on human SK-N-SH cells: scFvs I4 and I6 inhibited L1 functions, whereas ScFvs I13 and I27 triggered L1 functions *in vitro* (Table 1). L1 binds to integrins via the RGD motif in the sixth Ig-like domain to stimulate signal transduction resulting in cell adhesion and migration [26]–[29] and can recruit integrins in a non-RGD dependent manner via a dibasic sequence within the third Fn3 domain [30]. Furthermore, through homophilic trans-interactions or antibody cross-linking, L1 signaling can be induced, including calcium influx, scr phosphorylation and MAP kinase pathway activation [27]. Interaction of L1 with the fibroblast growth factor receptor (FGFR) was shown to be important for L1-mediated functions, such as neurite outgrowth [31], [32]. Binding of L1 to TAG-1, contactin and/or NCAM in cis-interactions may also be required for L1-L1 mediated neurite outgrowth from some neuronal cell types [33]–[38]. L1 interacts with CD24 in trans and, depending on the signaling complex formed with TAG-1, contactin or Caspr, neurite outgrowth is stimulated or inhibited [39]. L1 mediated cell adhesion can also depend on interaction with the epidermal growth factor receptor (EGFR) and signals via receptor tyrosine kinases [40]. Since src phosphorylation and MAP kinase activation are triggered by L1 signaling mediated by integrins, FGFR and L1-L1 homophilic interaction, we focused on these L1-triggered downstream signaling pathways and found that I4 and I6 reduced phospho-src and phospho-Erk1/2 levels, whereas I13 and I27 increased levels of phospho-src. This observation suggests that, depending on the epitope bound, L1 scFvs which block cell migration show a concomitant reduction of phospho-src, whereas scFvs which stimulate L1 functions, such as neurite extension, promote activation of src/Erk by phosphorylation. It is possible that normal neurons differ in their responses from neuronal tumor cells, but because of lack of access to such cells, this question could not be investigated.

**Table 1:** Binding properties and effects of the different scFvs.

ScFv	binding to	proliferation/migration	neurite outgrowth	cell survival	Src, ERK1/2 phosphorylation
I4	Ig1–4	−	−	−	−
I6	Ig1–4	−	−	−	−
I13	FN1–3	+	+	+	+
I27	FN1–3	+	+	+	+

Effect: inhibition (−), stimulation (+).

**Figure 9:**
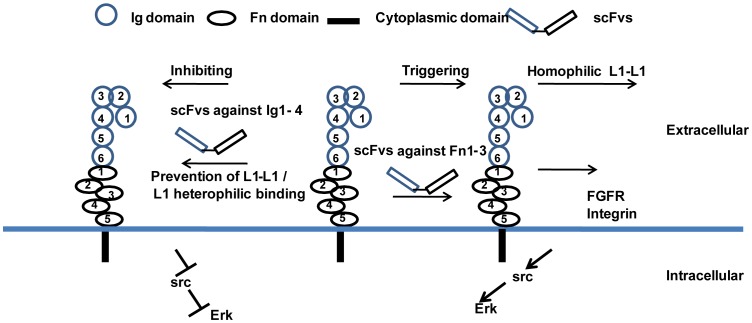
Signal transduction events following exposure of neuroblastoma cells to scFvs reacting with L1. ScFvs binding to the Ig1–4 domain of L1 in neuroblastoma cells inhibit phosphorylation of src and Erk most likely through disruption of homophilic or heterophilic L1 interactions. ScFvs binding to the Fn1–3 domain (such as the Fn3 domain of L1) increase phosphorylation of src and Erk by triggering of L1-L1 or L1-FGFR/L1-integrin signaling.

The Ig-like domains of L1 can take part in homophilic and heterophilic interactions between cells in cis- and trans-configurations. The six Ig-like domains have been suggested to form zipper-like adhesions between cells to support L1-L1 interaction at the cell surface [26], [41], [42], [43]. Domains Ig1–4 containing horse-shoe like structures are critical for L1 homophilic binding and promotion of neural cell growth and differentiation [25], [42]. The sixth Ig domain contains an RGD motif that binds to integrins to enhance L1-mediated migration [44], proliferation [45], and adhesion [16]. Binding of I4 and I6 to SK-N-SH cells results in reducing both proliferation and cell viability as well as migration through inhibition of phosphorylation of src and Erk, possibly through interruption of binding of L1 to integrins, such as alphaV beta3 and alpha9 beta1 integrin, of binding of L1 to L1 or of L1 homophilic interactions (Fig. 9) [26], [28], [29], [32]. These results are compatible with previous studies showing that interference with the L1-integrin association leads to disruption of interactions between axons and Schwann cells in peripheral nerves of mice [45] and to L1-mediated deterioration of blood vessels [46]. Rotary shadowing studies on L1 showed that the Fn domains form a conserved globular structure at the cell surface [47] and that interactions of L1 with homophilic or heterophilic partners, such as integrins, may change the conformation of the Fn domains. This ligand–induced change in conformation has been suggested by studies showing that a plasmin-sensitive peptide in domain Fn3 enhances homo-multimerization of L1 and recruitment of integrins, followed by signal transduction [30]. In the present study, I13 and I27 bind to the Fn1–3 domain of L1 resulting in increased cell survival and neurite outgrowth, correlating positively with the increase of proliferating cells. ScFvs that increased cell survival and neurite outgrowth also enhanced levels of phospho-src, phospho-Erk1/2, protected cells from hydrogen peroxide-induced cell death through Bcl-2, most likely as part of a signal transduction pathway downstream of L1–integrin interactions.

### Functional consequences

L1 is a multifunctional molecule in both the nervous system and malignant cells [18], [48]. We focused on scFvs against L1/ecd and characterized their inhibiting or triggering functions in the human neuroblastoma SK-N-SH cell line which expresses high levels of L1 and serves as an alternative to primary neural cells from human which are not readily available. Conventional L1 monoclonal antibodies have shown anti-tumor efficacy in human tumor xenograft models, thus encouraging their use in clinical diagnosis and treatment of L1-expressing tumors: Murine antibodies directed against the Ig1–2 domain of human L1 reduce ovarian tumor metastasis and prolong survival time in ovarian tumor-bearing nude mice [49]. Similarly, scFvs against Ig1–2 [50] have shown anti-tumor efficacy against intrahepatic cholangiocarcinoma in a nude mouse model [51], [52]. Conversely, a monoclonal L1 antibody against the N-terminus of rodent Fn3 has been shown to promote neurite outgrowth and to increase intracellular levels of Ca^2+^ and turnover of inositol phosphates [25]. Phosphorylated extracellular signal regulated-kinase Erk was increased by a function triggering polyclonal L1 antibody, suggesting that Erk activation mediates L1-stimulated neurite outgrowth through src [53]. ScFvs against mouse Fn1–2 containing part of the N-terminus of Fn3 increased neuronal survival and enhanced neurite outgrowth in primary neuronal cell culture [24]. Furthermore, monoclonal antibody 557 reacting with the N-terminus of Fn3 enhanced regeneration after peripheral nerve injury [54], [55]. Together, our results support the view that scFvs against different domains of L1 can not only inhibit functions in tissue invasion and metastasis of L1-expressing malignant cells, but also trigger L1 functions and their downstream signal transduction pathways in therapeutic approaches.

### Conclusion

ScFvs targeting different domains act as inhibitors or promoters of L1 functions.

## Supporting Information

Figure S1 **Human L1 domain fragments used in this study.** SDS-PAGE of purified L1/ecd, L1/Ig1–4 and L1/Fn1–3. Proteins were produced in insect Sf9 cells, purified by Ni affinity chromatography and subjected to SDS-PAGE under reducing conditions followed by staining with Coomassie G-250. Western blot analysis was performed using an antibody against the V5 epitope as primary antibody. Control without primary antibody did not show any signal.
(TIF)
