# Supplementary figures and images for: Antibody Fragments Directed against Different Portions of the Human Neural Cell Adhesion Molecule L1 Act as Inhibitors or Activators of L1 Function
Source: PLoS One. 2012 Dec 18;7(12):e52404. doi: 10.1371/journal.pone.0052404 (PMC3525558; doi:10.1371/journal.pone.0052404)

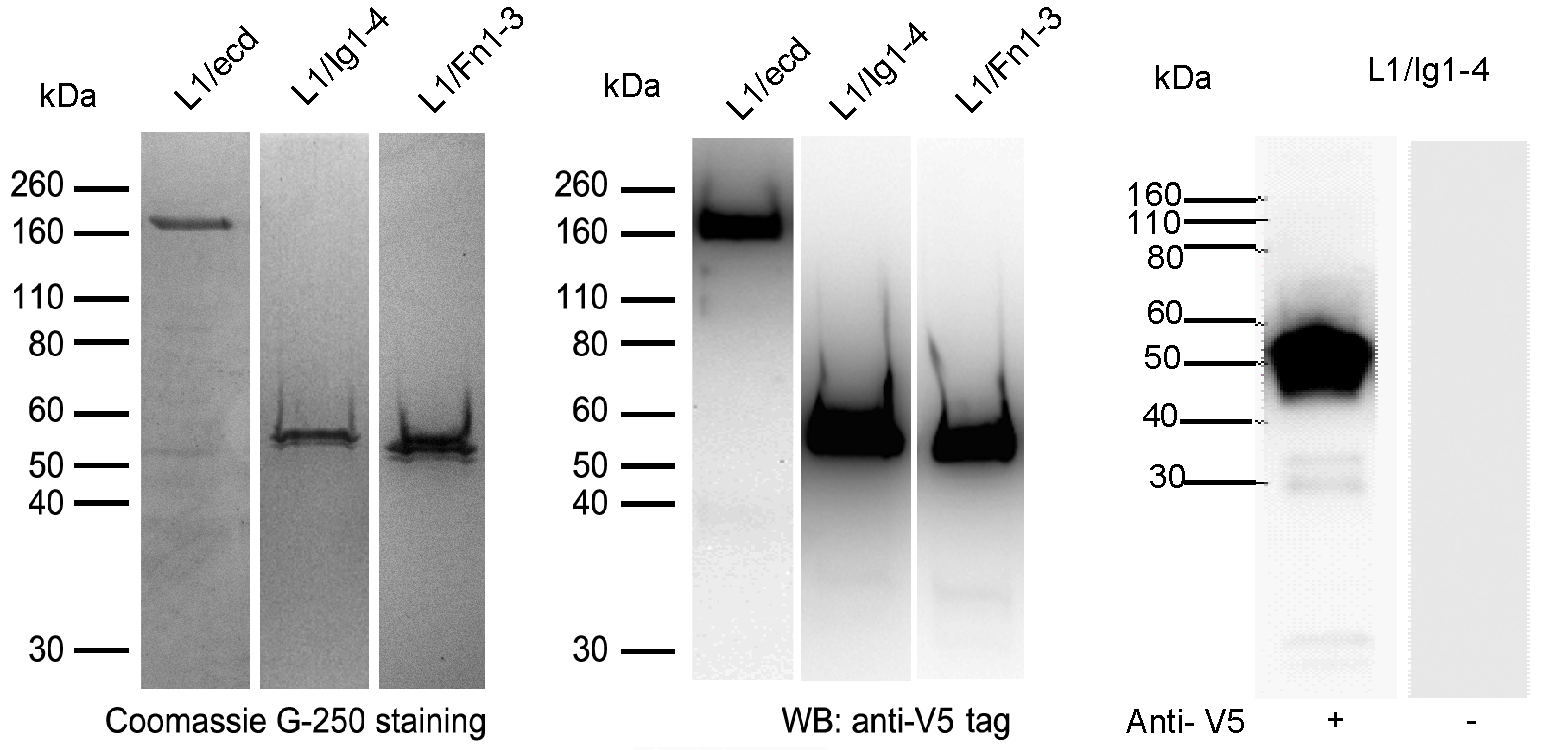

Supplement: Figure S1 — Human L1 domain fragments used in this study. SDS-PAGE of purified L1/ecd, L1/Ig1–4 and L1/Fn1–3. Proteins were produced in insect Sf9 cells, purified by Ni affinity chromatography and subjected to SDS-PAGE under reducing conditions followed by staining with Coomassie G-250. Western blot analysis was performed using an antibody against the V5 epitope as primary antibody. Control without primary antibody did not show any signal. (TIF) [file pone.0052404.s001.tif]
